# Experimental Study on the Detection of the Existence and Location of Mimicked and Unexpected Interface Debonding Defects in an Existing Rectangular CFST Column with PZT Materials

**DOI:** 10.3390/ma17133154

**Published:** 2024-06-27

**Authors:** Qian Liu, Bin Xu, Genda Chen, Weilong Ni, Zhixun Liu, Chun Lin, Zhiyou Zhuang

**Affiliations:** 1 School of Civil Engineering, Huaqiao University, Xiamen 361021, China; hqulq@stu.hqu.edu.cn; 2 Key Laboratory for Intelligent Infrastructure and Monitoring of Fujian Province, Huaqiao University, Xiamen 361021, China; 3 Department of Civil, Architectural and Environmental Engineering, Missouri University of Science and Technology, Rolla, MO 65401, USA; gchen@umsystem.edu; 4 Xiamen Holsin Engineering Testing Co., Ltd., Xiamen 361027, China; 5 C&D Holsin Engineering Consulting Co., Ltd., Xiamen 361015, China; lzx@holsin.cn (Z.L.); lc@holsin.cn (C.L.); 6 School of Civil Engineering, Beijing Jiaotong University, Beijing 100044, China; 21115037@bjtu.edu.cn

**Keywords:** piezoelectric lead zirconate titanate (PZT), concrete-filled steel tube (CFST) members, surface wave measurement, electromechanical impedance (EMI), interface debonding detection, experimental study

## Abstract

Interface bonding conditions between concrete and steel materials play key roles in ensuring the composite effect and load-carrying capacity of concrete–steel composite structures such as concrete-filled steel tube (CFST) members in practice. A method using both surface wave and electromechanical impedance (EMI) measurement for detecting the existence and the location of inaccessible interface debonding defects between the concrete core and steel tube in CFST members using piezoelectric lead zirconate titanate (PZT) patches as actuators and sensors is proposed. A rectangular CFST specimen with two artificially mimicked interface debonding defects was experimentally verified using PZT patches as the actuator and sensor. By comparing the surface wave measurement of PZT sensors at different surface wave travelling paths under both a continuous sinusoidal signal and a 10-period sinusoidal windowed signal, three potential interface debonding defects are quickly identified. Furthermore, the accurate locations of the three detected potential interface debonding defects are determined with the help of EMI measurements from a number of additional PZT sensors around the three potential interface debonding defects. Finally, the accuracy of the proposed interface debonding detection method is verified with a destructive observation by removing the local steel tube at the three detected interface debonding locations. The observation results show that the three detected interface debonding defects are two mimicked interface debonding defects, and an unexpected debonding defect occurred spontaneously due to concrete shrinkage in the past one and a half years before conducting the test. Results in this study indicate that the proposed method can be an efficient and accurate approach for the detection of unknown interface debonding defects in existing CFST members.

## 1. Introduction

Concrete-filled steel tube (CFST) members have been widely used in high-rise buildings, long-span bridges, and complex engineering projects due to their high load-carrying capacity, economic efficiency, convenient construction, and so on. However, due to the poor quality control approaches in concrete pouring technology and inevitable concrete shrinkage and creep in service, interface debonding defects between the concrete core and steel tube occur with high possibility, which directly affects their mechanical properties including load-carrying capacity and stiffness and seriously threatens their safety and serviceability [1,2,3,4]. Therefore, effective non-destructive testing (NDT) methods for interface debonding defect detection of existing CFST members is critical.

Traditional NDT methods including the hammer impact method, impact-echo method, and electromagnetic (EM) wave testing have been used to detect defects and damage in composite structures or concrete structures strengthened with composite materials, but they are usually ineffective in interface debonding defect detection in CFST members with a thick steel tube [5,6,7,8,9]. The hammer impact method for interface debonding detection is completely based on the experience of the operator, and no clear criteria are provided for the judgement. Therefore, the hammer impact method is a very rough quantitative method [5,6]. The EM wave method shows its abilities in the detection of interface debonding or cracks in concrete structures strengthened with carbon-fiber-reinforced polymer (CFRP) composite [7,8]. Unfortunately, the outer steel tube of CFST components acts as an electromagnetic shielding material and prevents the penetration of EM waves into the concrete core. Therefore, the interface debonding detection approach using the EM wave does not work for CFST members [9].

In recent decades, piezoelectric lead zirconate titanate (PZT) materials have been widely used for defect detection in engineering structures due to their low cost, good durability, and dual functions as either actuator or sensor. NDT technologies using PZT patches can be carried out mainly using either stress wave or electromechanical impedance (EMI) measurements and have been rapidly applied in civil engineering, aerospace engineering, and other fields [10,11,12,13]. Kocherla et al. [14] applied embedded PZT sensors to continuously monitor crack initiation in concrete structures using stress wave measurement. Moslehy et al. [15] used piezoelectric materials to monitor and evaluate the damage status of reinforced concrete (RC) columns under combined reversed cyclic loading. Markovic et al. [16] visually revealed stress wave propagation in concrete beams through finite element analysis and analyzed the influence of defects on it.

Aiming at interface debonding defect detection for concrete–steel composite structures including CFST members, Xu et al. [17,18,19] proposed a series of approaches based on stress wave measurements with surface-mounted or embedded PZT sensors. The feasibility of the proposed methods was verified experimentally and numerically with an assumption that a concrete core is a homogenous material. To efficiently explore the stress wave propagation mechanism, Xu and Luan et al. [20,21,22] established a two-dimensional time-domain spectral element method (SEM) for steel–concrete composite structures, and the effect of debonding defects on stress wave propagation within a coupled cross section substructure model was also explored. Results indicate the attenuation of the signal amplitude and energy of the stress wave measured by PZT sensors in the cross section of CFST members due to the existence of interface debonding defects. Considering the fact that the concrete core in CFST members is a typical material with heterogeneity and randomness at the meso-scale, Xu et al. [23] established a meso-scale multi-physics field coupling model composed of CFST and PZT to distinguish the influence of the heterogeneity and randomness of the concrete core in CFST members on stress wave propagation and that of interface debonding defects. Results showed that the interface debonding defect has the most significant influence on stress wave propagation in the cross section of CFST members and the output voltage signal of PZT sensors when compared with that of the heterogeneity and randomness of the meso-scale structure of the concrete core. For improving the computational efficiency of stress wave propagation in the CFST meso-model, Wang et al. [24] proposed a meso-scale equivalent homogenization method for CFST finite element models. The feasibility of the proposed method was verified numerically and experimentally. In practice, it is hard to embed PZT actuators or sensors into the concrete of an existing CFST member; it is easier to make surface wave measurements with surface-mounted PZT patches for interface debonding defect detection in CFST members [25]. Surface wave measurement was conducted by Xu et al. and Chen et al. [26,27,28] to identify interface debonding defects in CFST members, and its feasibility was verified experimentally and numerically with CFST members or cross sections with and without interface debonding defects. A crack detection method using Lamb wave measurements from PZT sensors for CFRP structures under static load was proposed by Ma et al. [29] Even the existing interface debonding detection approach is efficient in judging the existence of debonding, and further efforts are required in identifying the location or region of debonding defects.

Different from the stress-wave-based defect detection approach, electromechanical impedance (EMI) technology utilizes the piezoelectric coupling characteristics of the PZT sensor and the tested host structure to identify and locate local minor damage. The EMI technology has been successfully applied in many engineering fields as a local defect detection approach [30,31]. The feasibility of EMI-based detection technology has been verified for the detection of various structural damages such as crack development [32,33,34], metal corrosion [35], bolt loosening [36,37], post-earthquake pipeline damage assessment [38,39], and defects in concrete members [40,41,42,43,44]. Kuang et al. [45] proposed an approach for detecting damage in steel bars through EMI measurements. Nguyen et al. [46] established a multi-physical field coupling model composed of piezoelectric materials and host structures and numerically investigated its feasibility for anchoring force monitoring based on EMI analysis. Su et al. [47] used EMI technology to monitor the mechanical properties of mortar in concrete in real- time. In order to investigate the influence of temperature on EMI measurement, Baptista et al. [48] carried out experiments to investigate the variation in the peak frequency of the impedance signal over a temperature range of 25 °C to 102 °C. The results highlight that the correlation coefficient is effective for temperature compensation. Fan et al. [49] proposed the sparse regularization void defect detection method for plate structures based on EMI measurement under different temperature conditions. Additionally, critical load detection approaches for RC beams and RC beam–column joints based on EMI measurements have been proposed [50,51]. Kaur et al. [52] monitored the crack development of RC beams under four-point loading based on the EMI technique and explored the correlation between crack length, crack width, and distance from PZT sensors with the RMSD index. Furthermore, Ai et al. [53] have combined EMI measurements with a probability-weighted algorithm to locate the position of cracks in RC slabs.

To date, in most of the numerical and experimental studies on the feasibility of damage or defect detection approaches for engineering structures, the damage or defect is usually known before detection. In practice, the existence and the location of most inner defects such as interface debonding between a steel tube and concrete core are unknown or inaccessible. Therefore, it is of utmost importance to present an efficient interface debonding defect detection and location method for CFST members in practical applications.

In this study, combining surface wave measurement and EMI technology, an NDT method suitable for the identification of both the existence and location of unknown interface debonding defects in rectangular CFST members was proposed. Its feasibility was experimentally verified with mimicked interface debonding defects and unexpected interface debonding defects that are unknown before the test. Test results show that, based on the surface wave measurement using one pitch and one catch (OPOC) configuration, the range of unknown debonding defects can be determined by the amplitude variation in the output signal of PZT sensors under the excitation of a continuous sinusoidal signal and a 10-period sinusoidal windowed signal. After the ranges of potential interface debonding defects are determined, EMI measurements are conducted on additional PZT sensors around the estimated debonding regions. Finally, the accuracy of the proposed interface debonding defect detection and location method is validated with a destructive observation at the detected defect locations. In total, three interface debonding defects were detected. Two artificially mimicked interface debonding defects in the CFST specimen and one unexpected interface debonding defect were detected and localized successfully. The unexpected interface debonding defect might have occurred spontaneously due to concrete shrinkage in the specimen that had been placed in a lab for one and a half year. Results indicate that the proposed method can efficiently and accurately identify the existence and the location of the mimicked and unexpected interface debonding defects of CFST members, showing its applicability in practice.

This article is divided into five parts. The second part briefly introduces the interface debonding detection principles for existing CFST members with surface wave and EMI measurement. The third part shows the detection results of the regions of three potential interface debonding based on surface wave measurement methods. Then, in the fourth part, the location of each potential interface debonding defect is accurately located by EMI measurement from additional PZT sensors. Therefore, in the fifth part, the location of the detected three defects is verified by a destructive observation. Finally, the conclusions are summarized.

## 2. Principle of Interface Debonding Defect Detection with Surface Wave and EMI Measurements

### 2.1. Detection of Interface Debonding along the Surface Wave Propagation Path from a Surface Mounted PZT Actuator to a Sensor

**Figure 1:**
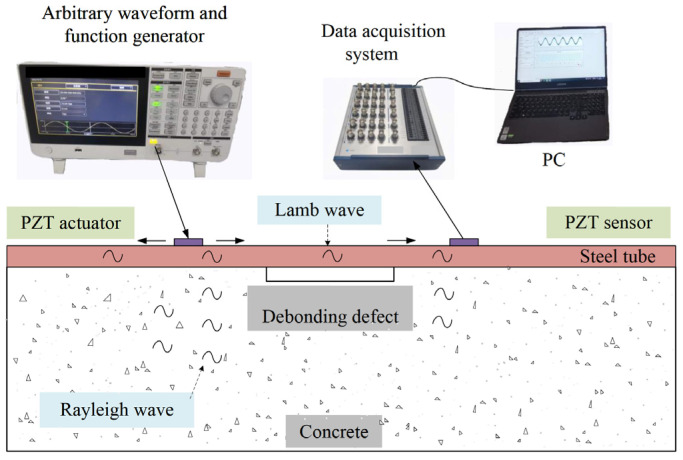
Propagation characteristics of stress waves in CFST based on stress wave detection method.

For interface debonding defect detection in a rectangular CFST specimen based on surface wave measurements, a one pitch and one catch (OPOC) configuration is employed as shown in Figure 1. A PZT patch pasted on the outer surface of the steel tube of the CFST specimen to be tested works as the actuator, and another PZT patch mounted on the outer surface of the steel tube is treated as the sensor to measure the surface wave transferring from the PZT actuator. The signal generated by the function generator is used to excite the PZT actuator. The response of the PZT sensor is recorded with a data acquisition system.

If no interface debonding defect exists between the PZT actuator and sensor, the stress wave propagates along the steel tube in the form of Rayleigh waves and simultaneously into the concrete core in both longitudinal and shear waves. When an interface debonding defect occurs between the PZT actuator and sensor, the Rayleigh wave in the steel tube transforms into a Lamb wave at the beginning of the interface debonding defect. The Lamb wave propagates along the steel tube with debonding and transfers to a Rayleigh wave again at the end of the interface debonding defect. The energy of stress waves propagating from the steel tube to the concrete core reduces when an interface debonding defect exists, which results in a higher energy of stress wave propagating along the steel tube when compared with that of a specimen without an interface debonding [23,54]. The variation in amplitude or energy of the surface wave measured by the PZT sensor provides an indicator of potential interface debonding defects along the surface wave travelling path from the PZT actuator to the PZT sensor.

### 2.2. Interface Debonding Localization Using EMI as a Local Method

The interface debonding defects within a CFST structure leads to a local stiffness reduction, resulting in a change in local mechanical impedance. However, the local mechanical impedance of an engineering structure is difficult to be measured directly. The impedance of a PZT sensor can be measured directly with the help of an impedance analyzer. Therefore, PZT sensors mounted on the outer surface of the CFST specimen can be used to detect the interface debonding defects. In this study, after detecting the existence of interface debonding defects in a certain surface wave propagation path, EMI measurements are made to localize the interface debonding defects.

**Figure 2:**
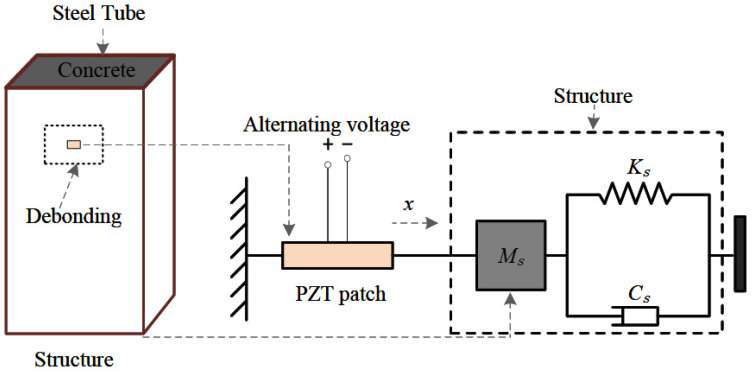
EMI measuring system for 1D PZT-structure coupling model.

Figure 2 shows the principle for interface debonding defect localization identification based on EMI measurement. In the coupling system composed of a PZT sensor and the host structure of CFST, the PZT sensor is mounted on the outer surface of the CFST structure by epoxy resin. Due to the inverse piezoelectric effect, the voltage signal produced by an impedance analyzer excites the PZT patch and the host structure of CFST along its thickness direction. At the same time, due to the positive piezoelectric effect, the dynamic response of the host structure is converted into an electrical signal through the PZT patch. Then, the EMI signature of the PZT patch can be captured by the impedance analyzer. In the coupled system composed of the host structure of CFST and the PZT patch, if all the parameters of the PZT sensor remain unchanged, the EMI signature of the PZT output is only affected by the stiffness of the host structure. Therefore, the defects in the host structure of the CFST can be determined by comparing and analyzing the difference in EMI signatures from PZT patches located at the defect locations and at healthy locations.

A one-dimensional PZT-structure coupling system was proposed by Liang [12], where only the axial compression deformation of the PZT patch and the interactions between the PZT patch and the host structure of CFST along the thickness direction of the PZT patch are considered. The electrical admittance Y of the EMI signal of the surface-mounted PZT sensor can be expressed as Equation (1).

(1) $$Y=\frac{1}{Z_{\omega }}=i\omega \frac{w_{A}l_{A}}{h_{A}}[\varepsilon_{33}^{T}(1-i\delta )-\frac{Z_{S}}{Z_{A}+Z_{S}}d_{31}^{2}{\bar{Y}}_{11}^{E}],$$

where *i* is the virtual unit, ω is the excitation angular frequency, $w_{A}$, $l_{A}$, and $h_{A}$ are the width, length, and thickness of the PZT patch mounted on the outer surface of the CFST specimen, respectively. $\varepsilon_{33}^{T}$, ${\bar{Y}}_{11}^{E}$ are the complex dielectric constant and complex Young’s modulus of the PZT patch at a constant electric field. $d_{31}$, δ are the piezoelectric constant and dielectric loss factor of the PZT patch, respectively. $Z_{A}$, $Z_{S}$ are the mechanical impedance of the PZT patch and the host structure of CFST, respectively. Equation (1) shows that the mechanical impedance value $Z_{S}$ of the host structure of CFST and the PZT material properties (including $Z_{A}$) determine the electrical impedance $Z_{\omega }$ completely when the PZT material parameters are constant, and the change in electrical impedance $Z_{\omega }$ indirectly expresses the change in mechanical impedance value $Z_{S}$. In this study, detailed parameters of the PZT material can be found in Table 1.

EMI-based interface debonding defect detection methods usually use voltage excitation in a high frequency range, so it is very sensitive to local minor damage such as interface debonding defects in CFST specimens. Therefore, as a typical local approach, the EMI-based interface debonding detection approach can be helpful for locating the interface debonding after detecting the surface wave’s travelling path with debonding defects using surface wave measurement as described above.

## 3. Experimental Study on Preliminary Interface Debonding Detection with Surface Wave Measurement

### 3.1. Rectangular CFST Specimen and Experiment Scheme

**Figure 3:**
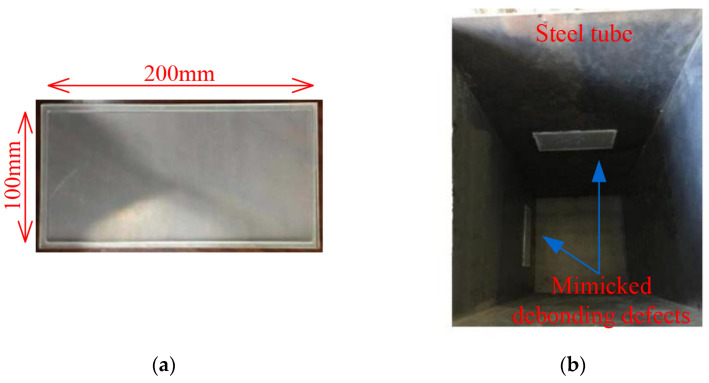
Artificially mimicked interface debonding defects in CFST specimen (**a**) Acrylic plate with groove; (**b**) Mimicked interfacial debonding defects.

To mimic interface debonding defects occurring in CFST members in practice, acrylic plates with grooves of specified dimensions are first fabricated and then installed at specific locations of the inner surface of the steel tube with epoxy resin before the concrete pouring. The material and installation of artificially mimicked interface debonding defects are shown in Figure 3a,b. An interface debonding defect with a height of 100 mm, width of 200 mm, and depth of 3 mm is mimicked on two inner surfaces of the steel tube of the specimen, as shown in Figure 3b. The remaining two inner surfaces do not have artificially mimicked interface debonding defects.

**Figure 4:**
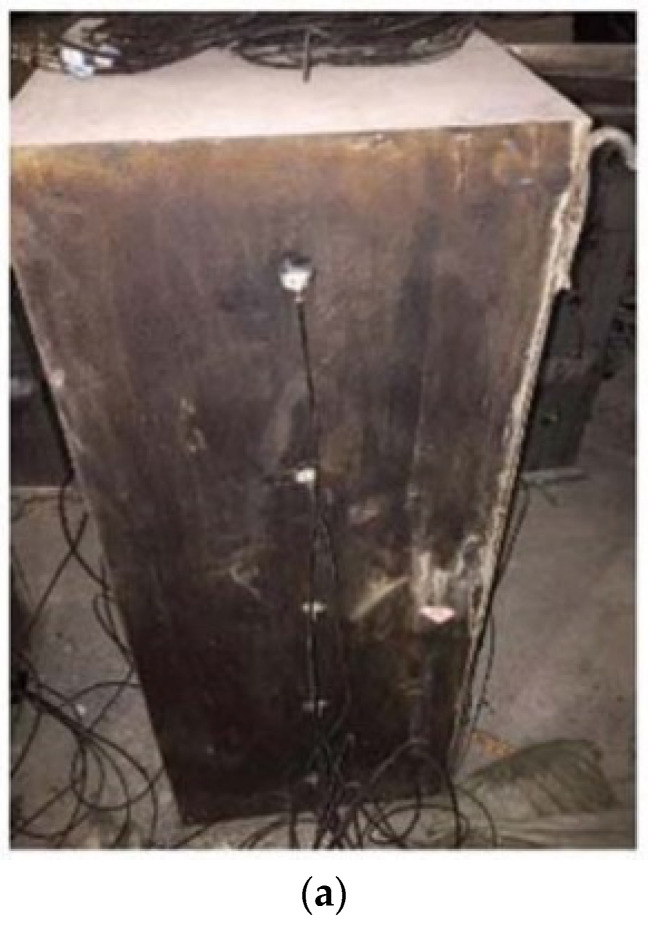

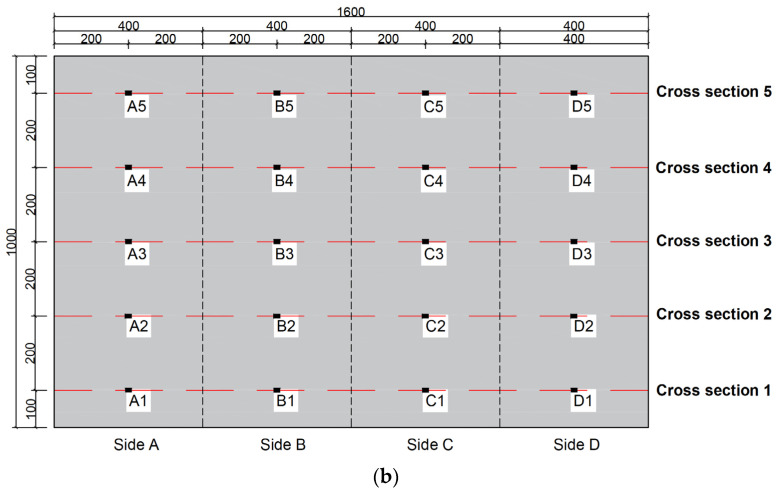
The rectangular CFST column and arrangement of PZT sensors (unit: mm). (**a**) Rectangular CFST member; (**b**) Arrangement of PZT sensors.

To validate the method proposed in this study for the detection of interface debonding defects and its localization, an interface debonding defect detection experiment is designed. Figure 4a shows the rectangular CFST specimen with surface-mounted PZT patches. The rectangular CFST member has a side length of 400 mm and a height of 1000 mm. The steel tube, made of Q235 grade steel with a thickness of 5 mm, is to be filled with concrete core of strength grade C30. The CFST specimen is divided into five cross sections at an interval of 200 mm in the vertical direction, labelled cross Section 1 to cross Section 5. Five PZT-5A patches are mounted on the four outer surfaces of the specimen, marked as A1~A5, B1~B5, C1~C5, and D1~D5 on Side A, B, C, and D, respectively. The arrangement of the sensors is shown in Figure 4b. It is worth noting that, before testing, the two sides with mimicked interface debonding defects are unknown.

### 3.2. Excitation Signals

**Figure 5:**
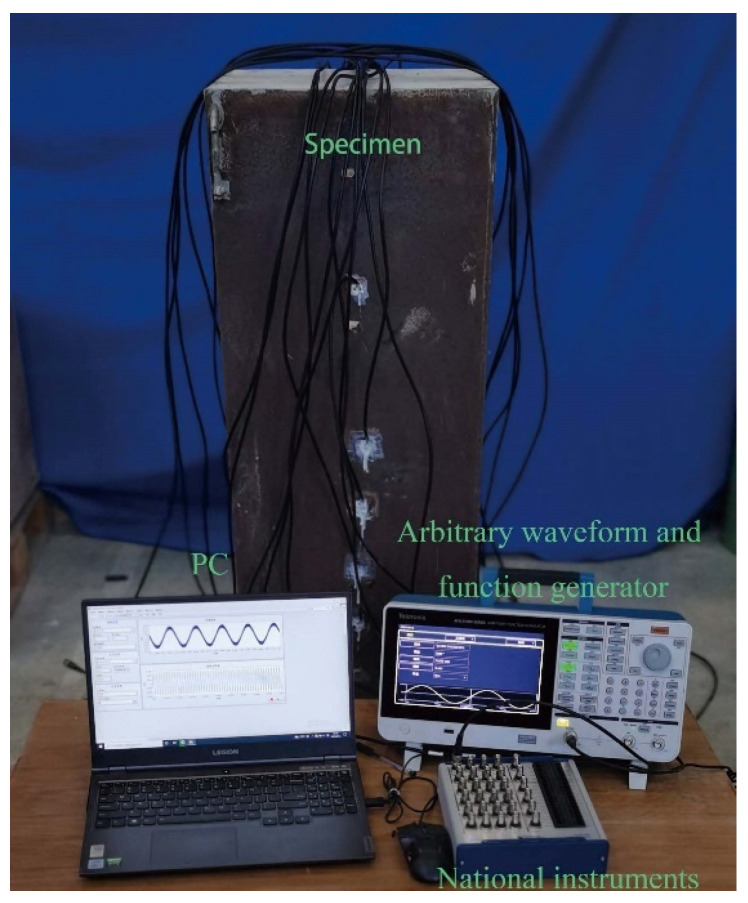
Test system for surface wave measurement.

To investigate the feasibility of the proposed interface debonding defect detection and localization approach in the CFST specimen, surface wave measurements by PZT sensors at different wave traveling paths were made and used to determine the regions of potential defects. Figure 5 shows the test setup. An arbitrary function generator is employed to drive a PZT actuator, while concurrently capturing the output signal of a surface-mounted PZT sensor via a data acquisition instrument.

The frequency selection of an excitation signal directly affects the accuracy of damage identification. Xu et al. and Chen et al. illustrated the selection criteria of excitation signals for the interface debonding defect detection of CFST members [26,27,28]. When the frequency of the excitation signal is low, the wavelength of the stress wave may exceed the dimension of the structure, resulting in a serious diffraction phenomenon, which leads to the disorder of voltage signals collected by the PZT sensors and further affects the interface debonding defect detection ability. When the frequency of the excitation signal is too high, the energy of the stress wave decays faster, and the interface debonding detection result is poor [28]. Considering the stress wave propagation mechanism and attenuation characteristics in the concrete core and steel tube of the CFST member to be investigated in this study, continuous sinusoidal signals and 10-phase sinusoidal window signals with an amplitude of 10 V and a frequency of 20 kHz are used as excitation signals.

### 3.3. Analysis of Output Voltage of Surface-Mounted PZT Sensor Measurements

In this test, a waveform signal generator is used as the signal excitation terminal. The electrical signals of PZT sensors are collected through a dynamic data acquisition system, and then, the electrical signals of PZT sensors of different surface wave travelling paths are analyzed to preliminarily identify the range of potential interface debonding defects. Here, for the convenience of expression, the surface wave travelling path is defined in the form of PZT actuator–PZT sensor.

**Figure 6:**
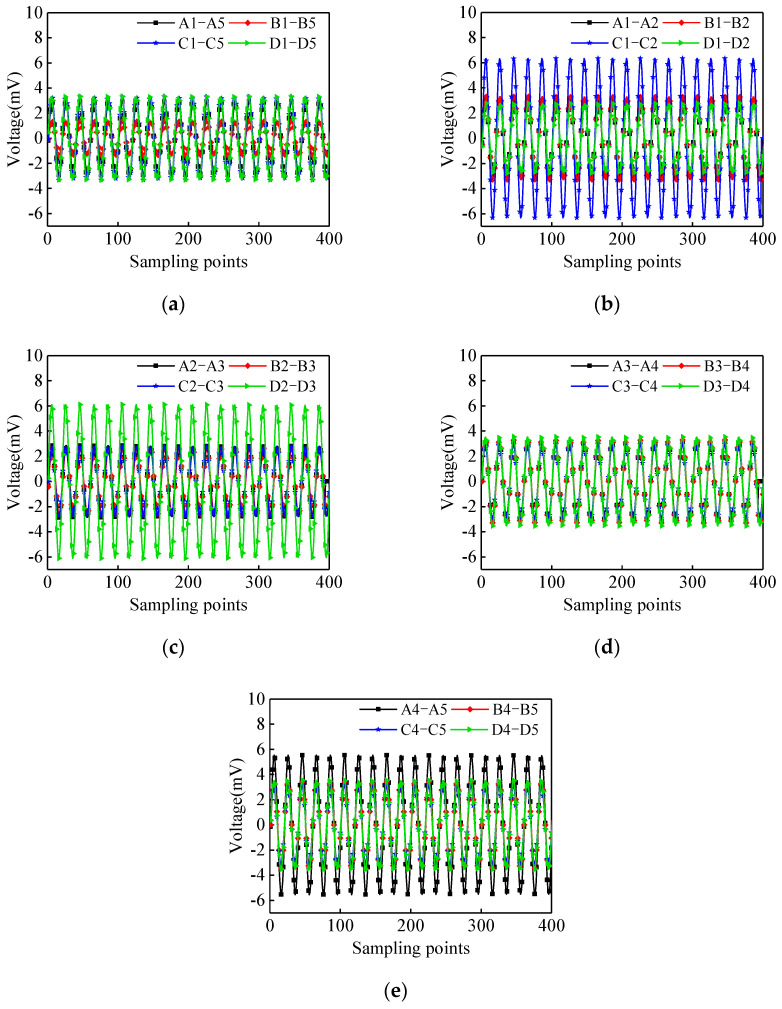
The output voltage signals corresponding to different surface wave travelling paths under continuous sinusoidal signal: (**a**) Path 1–5; (**b**) Path 1–2; (**c**) Path 2–3; (**d**) Path 3–4; (**e**) Path 4–5.

The output voltage signals corresponding to different surface wave travelling paths are shown in Figure 6. As shown in Figure 6a, under the continuous sinusoidal signal excitation, the output signal amplitude of the PZT sensors corresponding to the surface wave travelling paths of A1–A5, C1–C5, and D1–D5 are significantly higher than the output signal amplitudes of the PZT sensors corresponding to the traveling paths B1–B5. The existence of debonding defects will decrease the energy of the surface wave propagating into the concrete core, resulting in a higher signal amplitude of the PZT sensor when compared with that of CFST members without interface debonding defect. Therefore, it can be tentatively determined that there are interface debonding defects in Sides A, C, and D, and the bonding condition of Side B is good. From the analysis of the output voltage signal of each surface wave travelling path as shown in Figure 6b–e, the amplitude of the PZT sensors corresponding to the surface wave travelling paths C1–C2, D2–D3, and A4–A5 are larger than others with an identical measurement distance. The regions corresponding to the surface wave travelling paths of A4–A5, C1–C2, and D2–D3 are preliminarily detected as the regions with potential interface debonding defects. The output voltage amplitudes of PZT sensors of other surface wave travelling paths are very close, indicating that no interface debonding defects within the corresponding surface wave travelling paths can be detected.

**Figure 7:**
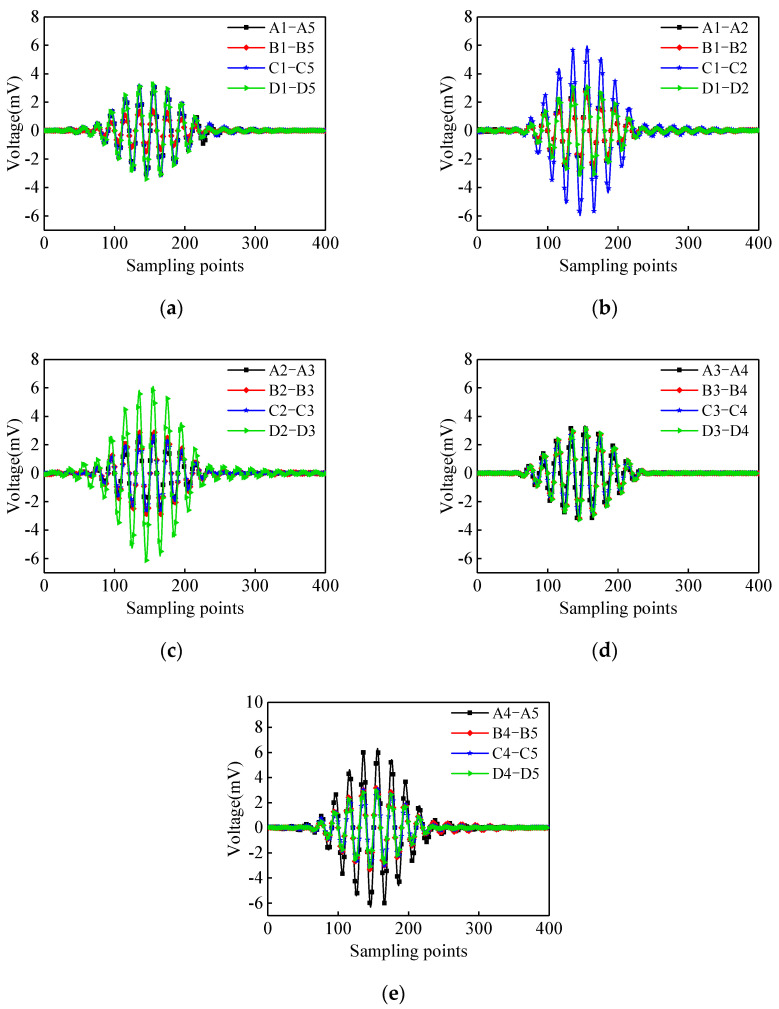
The output voltage signals corresponding to different surface wave travelling paths under a10-period sinusoidal windowed signal: (**a**) Path 1–5; (**b**) Path 1–2; (**c**) Path 2–3; (**d**) Path 3–4; (**e**) Path 4–5.

The output voltage signals of the surface-mounted PZT sensors excited by the 10-period sinusoidal windowed signal are shown in Figure 7. The output signal amplitude of the surface-mounted PZT sensors corresponding to the surface wave travelling paths of A1–A5, C1–C5, and D1–D5 are significantly higher than those of the path of B1–B5. Further measurements reveal that the output signal amplitude of the paths of C1–C2, D2–D3, and A4–A5 is also significantly higher than those of the remaining paths with an identical measurement distance. The results are consistent with the results of the surface wave measurements under the continuous sinusoidal excitation signals described above.

According to the propagation law of surface waves, the existence of interface debonding defects will significantly reduce the propagation of surface wave energy from the steel tube to the concrete. It can be seen from the test results that the amplitude change in the output signal of the PZT sensor can be used to effectively detect the existence of the unknown interface debonding defect of the CFST member and to preliminarily determine the location of protentional interface debonding defects under the excitation of the continuous sinusoidal signal and the 10-period sinusoidal windowed signal.

## 4. Experimental Study for Detailed Interface Debonding Localization Detection with EMI Measurements

### 4.1. EMI Measurement System

**Figure 8:**
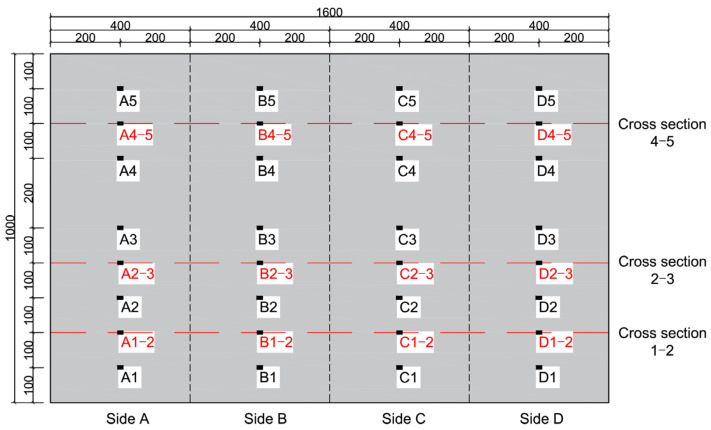
Additional PZT sensors arrangement.

**Figure 9:**
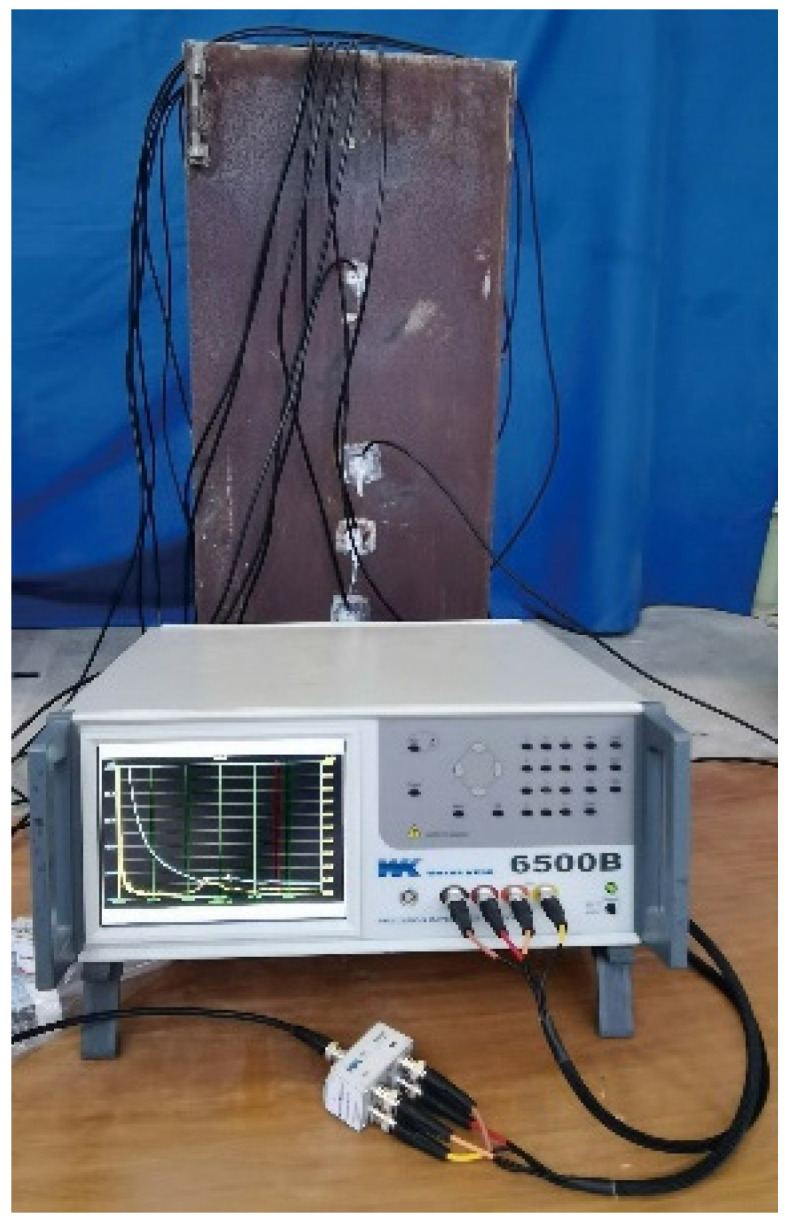
Test setup based on EMI technology.

To more precisely localize the detected interface defects, EMI measurement is carried out. According to the results of the surface-wave-measurement-based interface debonding detection, additional PZT sensors are installed at the center of the surface wave travelling paths of 1–2, 2–3, and 4–5 on the Sides A, B, C, and D, and the additional PZT sensors are marked in red. The position and label of PZT sensors are shown in Figure 8. The impedance analyzer is used to apply high-frequency voltage excitation to PZT sensors for EMI measurement, and the impedance signals are obtained using the spontaneous and self-receiving characteristics of PZT sensors. The interfacial bonding state of the CFST specimen is judged by comparing and analyzing the impedance signals of the PZT sensors at different locations. Figure 9 shows the test setup of interface debonding defect detection based on the EMI technology.

**Figure 10:**
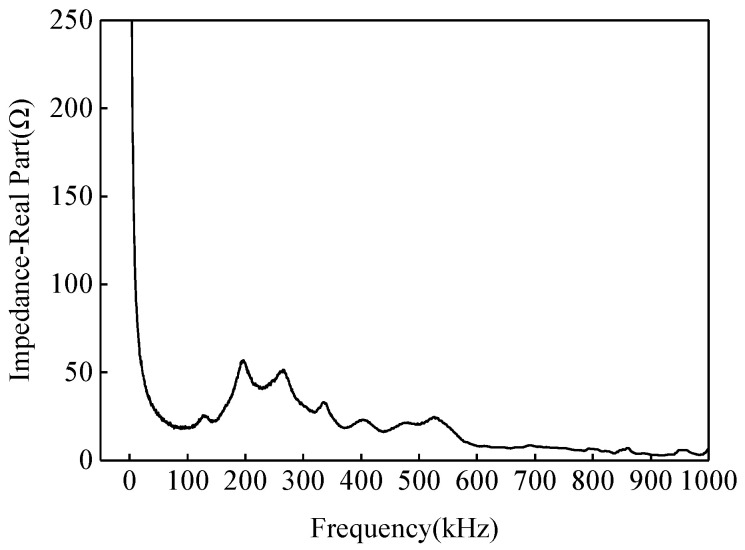
Selection of impedance frequency band (B4–5).

In the interface debonding localization approach with EMI measurements, it is important to select an appropriate frequency range. If the excitation signal frequency is too high, the EMI measurement is more sensitive to the environment temperature, bonding layer between PZT sensors and the steel tube, and boundary conditions of the specimen than to structural defects. Conversely, if the excitation signal frequency is too low, the EMI measurement is insensitive to minor defects such as interface debonding defects in CFST members [43,53,55]. In order to select a suitable frequency band for the interface debonding localization of the CFST specimen, an EMI measurement with a broad frequency band is made at first. Moreover, considering the fact that local defects such as interface debonding in a CFST member lead to local stiffness decrease, impedance curves within an appropriately selected frequency band should have locally dense peaks for the convenience of comparison. Figure 10 shows the EMI measurement of the PZT sensor B4–5 at the location without an interface debonding defect. It is clear that the impedance frequency curve between 100 kHz and 400 kHz has significant fluctuations and several peaks. Therefore, in this test, the EMI measurement within the frequency band between 100 kHz to 400 kHz is selected for the EMI measurement for each PZT sensor for the purpose of localizing the preliminarily detected interface debonding defects.

### 4.2. Impedance Signal Analysis of the CFST Specimen with Mimicked Interface Debonding Defects

To verify the effectiveness of the PZT-based EMI method for accurately localizing the interface debonding defect in the CFST specimen, EMI tests were conducted on the PZT sensor at identical heights of the four sides of the CFST specimen. The impedance was obtained using an impedance analyzer with a scan frequency range of 100 kHz–400 kHz. All measurement tests were carried out at room temperature around 25 °C.

**Figure 11:**
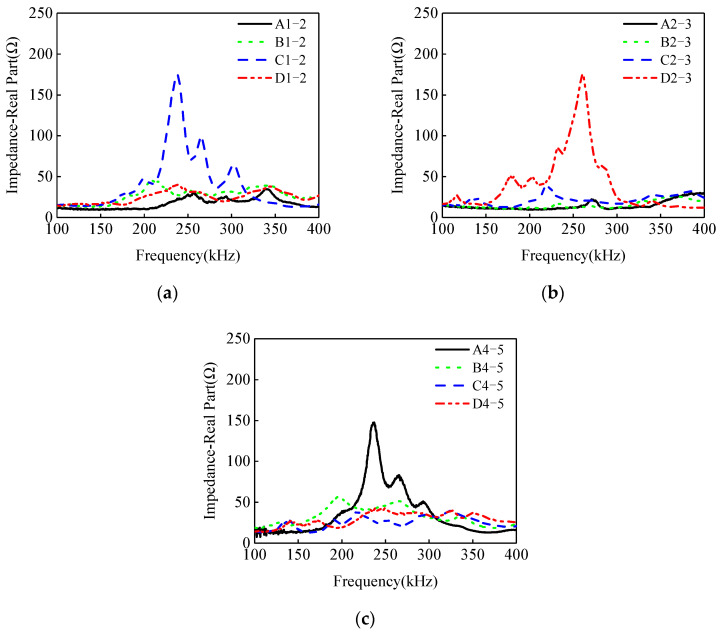
The impedance of PZT sensors at different measurement points: (**a**) Measurement points A1–2~D1–2; (**b**) Measurement points A2–3~D2–3; (**c**) Measurement points A4–5~D4–5.

The EMI measurements of PZT sensors are exhibited in Figure 11. It can be found that, within the EMI measurements from the four PZT sensors located at the cross sections 1 and 2, three impedance curves of the PZT sensors are close, but the EMI of measurement points C1–2 is obviously different from those of the other three PZT sensors measurements. A similar pattern has been observed in related experimental studies [11,56]. The reason is interface debonding leads to an obvious decrease in the local stiffness of the CFST specimen, resulting in a significant variation in the EMI signals of the PZT sensor located at the steel tube with interface debonding defects when compared to those from the PZT at the healthy location. The results show that the interface debonding on Side C is located at the location of PZT sensor C1–2. No interface debonding defects occur on other three sides at the cross sections 1 and 2. Similar findings can be made for the other two cross sections of 2–3 and 4–5. The interface debonding defect on Side D is located at the cross sections 2 and 3, and the interface debonding on Side A is located at cross sections 4 and 5.

Based on the analysis results of the EMI measurement of PZT sensors shown in Figure 11, the measurement points A4–5, C1–2, and D2–3 can be identified as locations with interface debonding defects.

**Figure 12:**
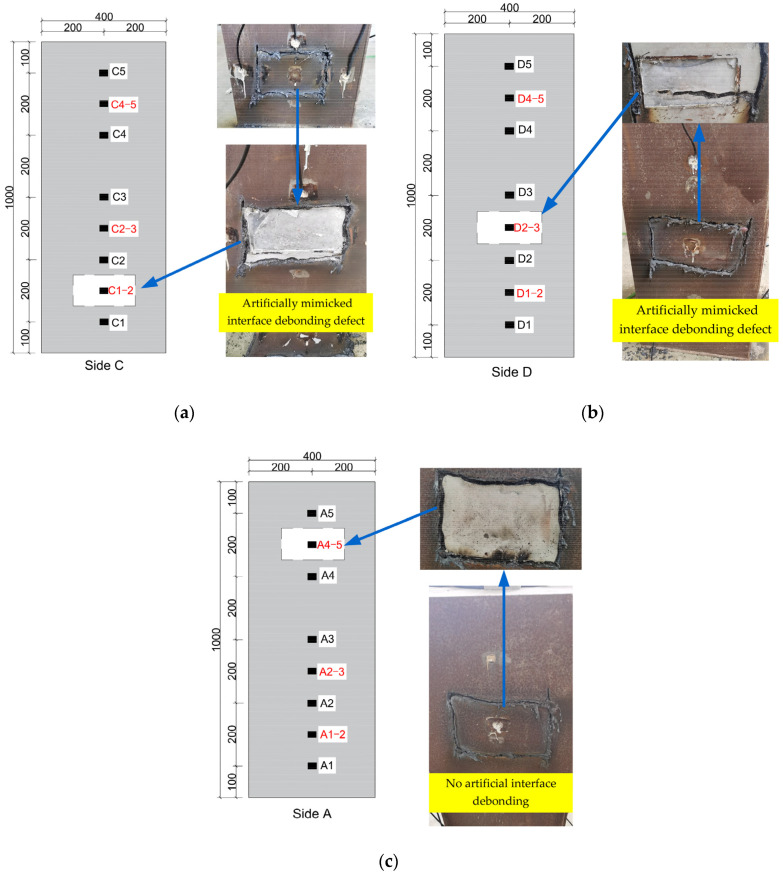
Destructive observation on the detected interface debonding defects by removing local steel tube treatment of detected defect locations: (**a**) Location of measurement point C1–2; (**b**) Location of measurement point D2–3; (**c**) Location of measurement point A4–5.

To verify the detection results about the interface debonding defect existence and localization, a destructive observation was carried out by cutting the steel tube around the detected debonding defects. After cutting the steel tube, the bonding state between the steel tube and the core concrete of the CFST specimen can be observed directly. Figure 12a,b show the observation results that the two mimicked interface debonding defects at the measurement points C1–2 and D2–3 were found, which is consistent with the artificially mimicked interface debonding defects before the test. At the measurement point A4–5, an interface debonding between the steel tube and concrete core was also observed. The steel plate fell off by itself after the four sides were cut, which indicates that no bonding existed between the steel tube and the steel tube at point A4–5 as shown in Figure 12c. Actually, no artificial interface debonding was made at point A4–5, and the reason for the initiation of the interface debonding at point A4–5 might be the shrinkage of the concrete core of the CFST specimen that had been placed in the lab for over one year. The above experimental studies demonstrate the effectiveness of the detection approach for CFST members with mimicked and unexpected interface debonding defects by the use of a combination of surface wave measurement and EMI technology.

## 5. Conclusions

This paper innovatively proposed a rapid, convenient, and effective NDT detection technique for the interface debonding defect in CFST members using PZT patches, and the feasibility of the proposed approach was validated experimentally with a rectangular CFST specimen with artificially mimicked and unexpected interface debonding defects. The locations of the mimicked defects were treated as unknown considering the fact that the existence and the locations of any possible interface debonding defects in any engineering structures in practice are unknown before testing. By combining surface wave measurements and EMI techniques, the unknown mimicked and unexpected interface debonding defects in the CFST members were accurately located. By analyzing the abnormal surface wave measurement, the regions with interface debonding defects were preliminarily determined, and then, the interface debonding defect locations were accurately determined with the help of the EMI measurements of PZT sensors arranged within the detected potential interface debonding regions. To verify the interface debonding defect detection results, the outer steel tube at the locations of the detected interface debonding defects were cut, and direct observation was carried out to check if artificially mimicked and unexpected interface debonding defects existed at the locations detected. The main conclusions can be drawn as follows.

- To quickly and efficiently judge the interface bonding condition and preliminarily determine the regions of interface debonding defects, surface wave measurements were made with the help of PZT sensors and actuators installed on the outer surface of the rectangular CFST specimen. Experimental results showed that the energy of the stress wave propagating from the steel tube to the concrete core decreased due to the existence of interface debonding between the PZT actuator and sensor. Therefore, the energy of the surface wave propagating in the steel tube between the PZT actuator and sensor in the surface wave travelling path with an interface debonding defect was greater than that without interface debonding defects under the excitation of either a continuous sinusoidal signal or a 10-period sinusoidal windowed signal. The existence of three interface debonding defects corresponding to three surface wave travelling paths, A4–A5, C1–C2, and D2–D3, were preliminarily identified by analyzing the changes in the signal amplitude of the surface wave measured by PZT sensors in different surface wave travelling paths.
- To further accurately determine the location of the unknown interface debonding defects in the specimen, additional surface-mounted PZT sensors were installed in the preliminary determined interface debonding defect regions for EMI measurement. The EMI measurements were compared, and the test results showed that the EMI curves at the measurement points of A4–5, C1–2, and D2–3 were significantly different from those at other measurement points at determined healthy regions. The EMI measurement at healthy regions were close to each other. The measurement points of A4–5, C1–2, and D2–3 can be judged as the locations of the mimicked defects.
- To verify the accuracy of the debonding defect detection results using both surface wave and EMI measurements for the rectangular CFST member with unknown interface debonding defects, a destructive observation was carried out by cutting the outer steel tube at the locations of the identified interface debonding defects. The observation results showed that actual interface debonding defects existed at the three detected interface debonding defect locations. Among the three detected interface debonding defects, the interface debonding defects at measurement points of C1–2 and D2–3 matched well with the actual artificially mimicked interface debonding defects, and the interface debonding defect at the measurement point A4–5 was unexpected because no artificially mimicked interface debonding was installed there. The existence of the defect at point A4–5 may have been caused by concrete shrinkage after the placement of the specimen in a lab for over one year.
- Experimental results showed that the proposed method can perform NDT for the detecting and localizing of interface debonding defects in rectangular CFST components rapidly and efficiently with PZT actuating and sensing technology. In practical engineering application, the dimensions of CFST columns are typically larger. On the basis of ensuring the accuracy of the interface debonding detection results, the layout of PZT sensors should be optimized. Moreover, a comprehensive investigation on the influence of internal ring plates and anchor bolts on stress wave propagation and EMI measurements should be carried out in the future.

## Figures and Tables

**Table 1:** Detailed parameters of the employed PZT patch.

Dimension		Material Parameters	
Width $w_{A}$	10 mm	Complex dielectric constant $\varepsilon_{33}^{T}$	1.77 × 10^−8^ F/m
Length $l_{A}$	15 mm	Complex Young’s modulus ${\bar{Y}}_{11}^{E}$	7.25 × 10^10^ N/m
Thickness $h_{A}$	0.3 mm	Piezoelectric constant $d_{31}$	185 PC/N
		Dielectric loss factor δ	1.5%

## Data Availability

Some or all data, models, or code that support the findings of this study are available from the corresponding author upon reasonable request.

## References

[1] Nematzadeh M., Fazli S., Naghipour M., Jalali J. (2017). Experimental study on modulus of elasticity of steel tube-confined concrete stub columns with active and passive confinement. Eng. Struct..

[2] Xue J., Zhang Y., Briseghella B., Chen B. Experimental research on effects of debonding on circular CFST columns with different slenderness ratios. Proceedings of the 9th International Conference on Arch Bridges.

[3] Wang X.L., Fan F.F., Lai J.X. (2022). Strength behavior of circular concrete-filled steel tube stub columns under axial compression: A review. Constr. Build. Mater..

[4] Wang F.C., Xie W.Q., Li B., Han L.H. (2022). Experimental study and design of bond behavior in concrete-filled steel tubes (CFST). Eng. Struct..

[5] Li L., Jiao J.P., Gao X., Jia Z.H., Wu B., He C.F. (2022). A review on nondestructive testing of bonding interface using nonlinear ultrasonic technique. Chin. Sci. B-Chin..

[6] Chen D.D., Wang L.W., Luo X.J., Fei C.L., Li D., Shan G.B., Yang Y.T. (2021). Recent development and perspectives of optimization design methods for piezoelectric ultrasonic transducers. Micromachines.

[7] Li Z., Haigh A.D., Saleh M.N., McCarthy E.D., Soutis C., Gibson A.A.P., Sloan R. (2018). Detection of impact damage in carbon fiber composites using an electromagnetic sensor. Res. Nondestruct. Eval..

[8] Moll J. (2020). Guided electromagnetic waves for damage detection and localization in metallic plates: Numerical and experimental results. Int. J. Microw. Wirel. Technol..

[9] Li D.S., Zhou J.L., Ou J.P. (2020). Damage, nondestructive evaluation and rehabilitation of FRP composite-RC structure: A review. Constr. Build. Mater..

[10] Hamzeloo S.R., Shamshirsaz M., Rezaei S.M. (2012). Damage detection on hollow cylinders by electro-mechanical impedance method: Experiments and finite element modeling. Comptes Rendus Mécanique.

[11] Liu Q., Xu B., Xia Z.J., Yao Y.D., Wang J. (2024). Interface debonding defect detection for CFST columns based on EMI measurements: Experiment, numerical simulation and blind inspection in practice. Adv. Struct. Eng..

[12] Liang C., Sun F.P., Rogers C.A. (1994). Coupled electro-mechanical analysis of adaptive material systems-determination of the actuator power consumption and system energy transfer. J. Intell. Mater. Syst. Struct..

[13] Selva P., Cherrier O., Budinger V., Lachaud F., Morlier J. (2013). Smart monitoring of aeronautical composites plates based on electromechanical impedance measurements and artificial neural networks. Eng. Struct..

[14] Kocherla A., Duddi M., Subramaniam K.V.L. (2021). Embedded PZT sensors for monitoring formation and crack opening in concrete structures. Measurement.

[15] Moslehy Y., Gu H.C., Belarbi A., Mo Y.L., Song G.B. (2010). Smart aggregate based damage detection of circular RC columns under cyclic combined loading. Smart. Mater. Struct..

[16] Markovic N., Nestorovic T., Stojic D. (2015). Numerical modeling of damage detection in concrete beams using piezoelectric patches. Mech. Res. Commun..

[17] Xu B., Li B., Song G.B. (2013). Active debonding detection for large rectangular CFSTs based on wavelet packet energy spectrum with piezoceramics. J. Struct. Eng..

[18] Xu B., Chen H.B., Xia S. (2017). Wave propagation simulation and its wavelet package analysis for debonding detection of circular CFST members. Smart. Struct. Syst..

[19] Xu B., Chen H.B., Mo Y.L., Chen X.M. (2017). Multi-physical field guided wave simulation for circular concrete-filled steel tubes coupled with piezoelectric patches considering debonding defects. Int. J. Solids Struct..

[20] Xu B., Luan L.L., Chen H.B., Ge H.B. (2018). Numerical study on interface debonding detection mechanisms with 2d spectral element method for concrete-filled steel tube using embedded PZT sensor. Smart Mater. Struct..

[21] Xu B., Luan L.L., Chen H.B., Wang H.D. (2020). Local wave propagation analysis in concrete-filled steel tube with spectral element method using absorbing layers—Part I: Approach and validation. Mech. Syst. Signal Proc..

[22] Luan L.L., Xu B., Chen H.B., Wang H.D. (2021). Local wave propagation analysis in concrete-filled steel tubes with spectral element method using absorbing layers—Part II: Application in coupling system. Mech. Syst. Signal Proc..

[23] Xu B., Chen H.B., Mo Y.L., Zhou T.M. (2018). Dominance of debonding defect of CFST on PZT sensor response considering the meso-scale structure of concrete with multi-scale simulation. Mech. Syst. Signal Proc..

[24] Wang J., Xu B., Liu Q., Guan R.Q., Ma X. (2023). Feasibility of stress wave-based debond defect detection for RCFSTs considering the influence of randomly distributed circular aggregates with mesoscale homogenization methodology. Materials.

[25] Lee F.W., Lim K.S., Chai H.K. (2016). Determination and extraction of Rayleigh-waves for concrete cracks characterization based on matched filtering of center of energy. J. Sound Vibr..

[26] Xu B., Luan L.L., Chen H.B., Wang J., Zheng W.T. (2019). Experimental study on active interface debonding detection for rectangular concrete-filled steel tubes with surface wave measurement. Sensors.

[27] Chen H.B., Xu B., Wang J., Luan L.L., Zhou T.M., Nie X., Mo Y.L. (2019). Interfacial debonding detection for rectangular CFST using the MASW method and its physical mechanism analysis at the meso-level. Sensors.

[28] Chen H.B., Xu B., Zhou T.M., Mo Y.L. (2019). Debonding detection for rectangular CFST using surface wave measurement: Test and multi-physical fields numerical simulation. Mech. Syst. Signal Proc..

[29] Ma X.S., Bian K., Lu J., Xiong K. (2016). Experimental research on detection for interface debond of CFRP T-joints under tensile load. Compos. Struct..

[30] Na W.S., Baek J. (2018). A review of the piezoelectric electromechanical impedance based structural health monitoring technique for engineering structures. Sensors.

[31] Fan X., Li J., Hao H. (2019). Impedance resonant frequency sensitivity based structural damage identification with sparse regularization: Experimental studies. Smart Mater. Struct..

[32] Liaqat A., Sikandar K., Naveed I., Salem B., Hamad H., Yong B. (2021). An experimental study of damage detection on typical joints of jackets platform based on electro-mechanical impedance technique. Materials.

[33] Zuo C.Y., Feng X., Zhang Y., Lu L., Zhou J. (2017). Crack detection in pipelines using multiple electromechanical impedance sensors. Smart Mater. Struct..

[34] Wang T., Tan B.H., Lu M.G., Zhang Z., Lu G.T. (2020). Piezoelectric electro-mechanical impedance (EMI) based structural crack monitoring. Appl. Sci..

[35] Li W.J., Wang J.J., Liu T.J., Luo M.Z. (2020). Electromechanical impedance instrumented circular piezoelectric-metal transducer for corrosion monitoring: Modeling and validation. Smart. Mater. Struct..

[36] Mascarenas D.L., Todd M.D., Park G., Farrar C.R. A miniaturized electromechanical impedance-based node for the wireless interrogation of structural health. Proceedings of the Health Monitoring and Smart Nondestructive Evaluation of Structural and Biological Systems.

[37] Le T.C., Ho D.D., Huynh T.C. (2021). Anchor force monitoring using impedance technique with single-point mount lead-zirconate-titanate interface: A feasibility study. Buildings.

[38] Park G., Cudney H.H., Inman D.J. (2001). Feasibility of using impedance-based damage assessment for pipeline structures. Earthq. Eng. Struct. Dyn..

[39] Na S., Tawie R., Lee H.K. (2012). Electromechanical impedance method of fiber-reinforced plastic adhesive joints in corrosive environment using a reusable piezoelectric device. J. Intel. Mat. Syst. Str..

[40] Ai D.M., Cheng J. (2023). A deep learning approach for electromechanical impedance based concrete structural damage quantification using two-dimensional convolutional neural network. Mech. Syst. Signal Proc..

[41] Kamal A., Shweta G., Sudhakara R.M. (2023). Crack healing in concrete by microbially induced calcium carbonate precipitation as assessed through electromechanical impedance technique. Eur. J. Environ. Civ. Eng..

[42] Ai D.M., Lin C., Zhu H.P. (2021). Embedded piezoelectric transducers based early-age hydration monitoring of cement concrete added with accelerator/retarder admixtures. J. Intel. Mat. Syst. Str..

[43] Tawie R., Lee H.K. (2010). Monitoring the strength development in concrete by EMI sensing technique. Constr. Build. Mater..

[44] Su Y.F., Han G.S., Nantung T., Lu N. (2020). Novel methodology on direct extraction of the strength information from cementitious materials using piezo-sensor based electromechanical impedance (EMI) method. Constr. Build. Mater..

[45] Kuang J., Xu B. Local damage detection for steel rebar by impedance measurements of PZT sensors. Proceedings of the Third International Conference on Smart Materials and Nanotechnology in Engineering.

[46] Nguyen T.H., Phan T.T.V., Le T.C., Ho D.D., Huynh T.C. (2021). Numerical simulation of single-point mount PZT-interface for admittance-based anchor force monitoring. Buildings.

[47] Su Y.F., Han G., Kong Z., Nantung T., Lu N. (2020). Embeddable piezoelectric sensors for strength gain monitoring of cementitious materials: The influence of coating materials. Eng. Sci..

[48] Baptista F.G., Budoya D.E., de Almeida V.A.D., Ulson J.A.C. (2014). An experimental study on the effect of temperature on piezoelectric sensors for impedance-based structural health monitoring. Sensors.

[49] Fan X., Li J. (2020). Damage identification in plate structures using sparse regularization based electromechanical impedance technique. Sensors.

[50] Voutetaki E.M., Papadopoulos A.N., Angeli M.G., Providakis C.P. (2016). Investigation of a new experimental method for damage assessment of RC beams failing in shear using piezoelectric transducers. Eng. Struct..

[51] Divsholi B.S., Yang Y.W., Bing L. (2009). Monitoring beam-column joint in concrete structures using piezo-impedance sensors. Adv. Mater. Res..

[52] Kaur H., Singla S. (2024). A Parametric study of the impact of crack parameters on EMI response in RC beams using correlation analysis. J. Vib. Eng. Technol..

[53] Ai D.M., Zhang D.L., Zhu H.P. (2024). Damage localization on reinforced concrete slab structure using electromechanical impedance technique and probability-weighted imaging algorithm. Constr. Build. Mater..

[54] Luan L.L., Xu B., Chen H.B. (2017). Numerical simulation on stress wave propagation of steel-concrete composite structures with interface debonding by spectral element method. Eng. Mech..

[55] Wang D.S., Song H.Y., Zhu H.P. (2013). Numerical and experimental studies on damage detection of a concrete beam based on PZT admittances and correlation coefficient. Constr. Build. Mater..

[56] Karayannis C.G., Golias E., Naoum M.C., Chalioris C.E. (2022). Efficacy and damage diagnosis of reinforced concrete columns and joints strengthened with FRP ropes using piezoelectric transducers. Sensors.

